# Supplementary material for: Bacteria can compensate the fitness costs of amplified resistance genes via a bypass mechanism
Source: Nat Commun. 2024 Mar 14;15:2333. doi: 10.1038/s41467-024-46571-7 (PMC10940297; doi:10.1038/s41467-024-46571-7)
Supplement: Supplementary file 1 — Supplementary Figs. and tables [file 41467_2024_46571_MOESM1_ESM.pdf]

Supplementary Fig. 1. Exponential growth rate of mutants at different antibiotic concentrations. (A) *E. coli* DA33135 selected on different concentrations of tobramycin. (B) *E. coli* DA33137 selected on different concentrations of gentamicin. (C) *K. pneumoniae* DA33140 selected on different concentrations of gentamicin. (D) *S.* Typhimurium DA34827 selected on different concentrations of tetracycline. Growth rate was measured from 6 biological replicates (n=6). Data are presented as mean values +/- standard deviation. The statistical testing was done using unpaired two-tailed Student's t test. The asterisks (*) indicate statistically significant (P < 0.01) changes in growth rate in the mutants compared to their respective wild-type strains. Exact P values are given in Source data file.

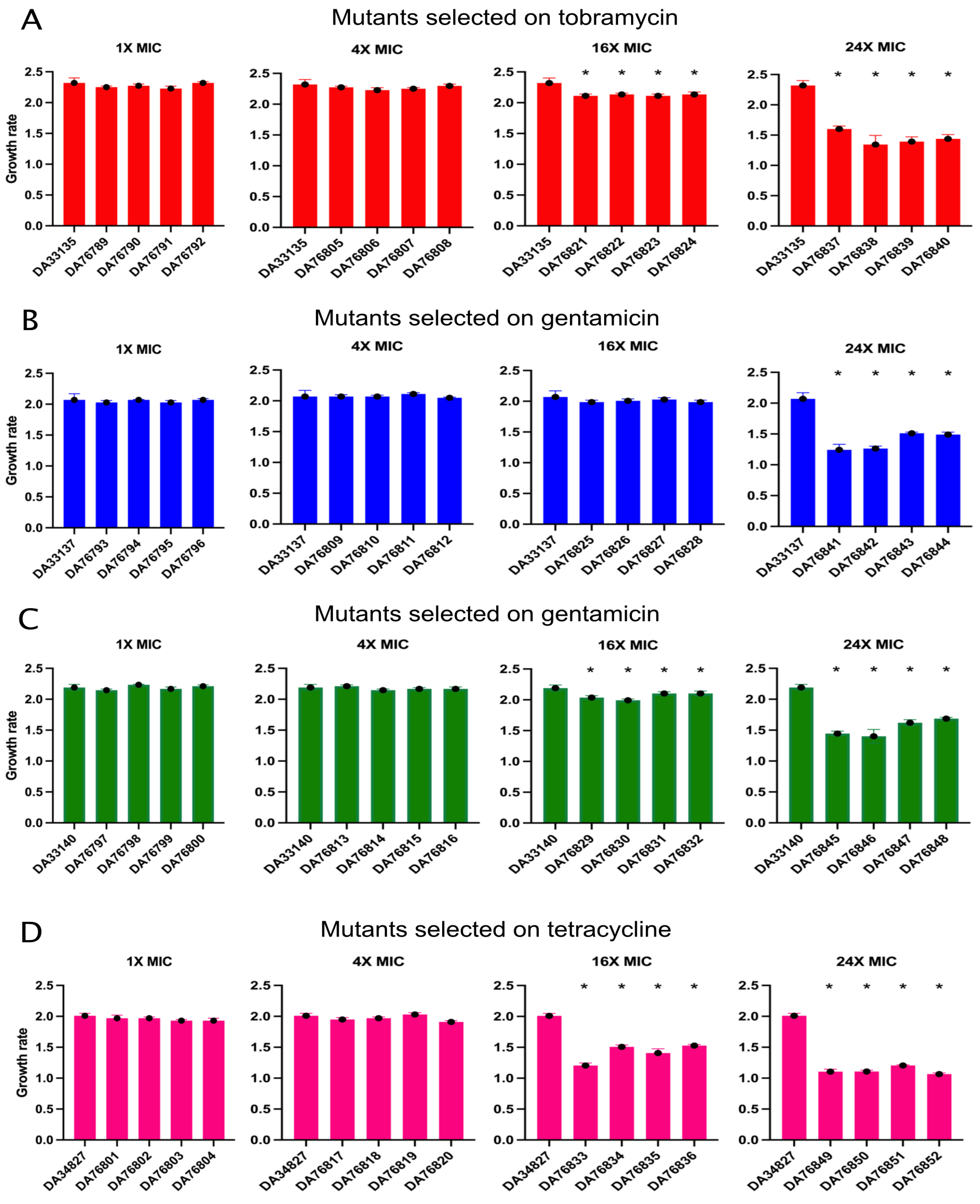

Supplementary Fig. 2. Exponential growth rate of compensated mutants after 100 generations. (A) *E. coli* mutants isolated after compensatory evolution on tobramycin. (B) *E. coli* mutants isolated after compensatory evolution on gentamicin. (C) *K. pneumoniae* mutants isolated after compensatory evolution on gentamicin. (D) *S.* Typhimurium mutants isolated after compensatory evolution on tetracycline. The black bars indicate the wild-type isolates. The dark colored bars indicate the parental resistant mutants isolated at 24X MIC and the light-colored bars indicate the compensated mutants. Growth rate was measured from 6 biological replicates (n=6). Data are presented as mean values +/- standard deviation. The statistical testing was done using unpaired two-tailed Student's t test. The asterisks (*) indicate statistically significant (P < 0.01) changes in growth rate in the mutants compared to their respective parental strains. Exact P values are given in Source data file.

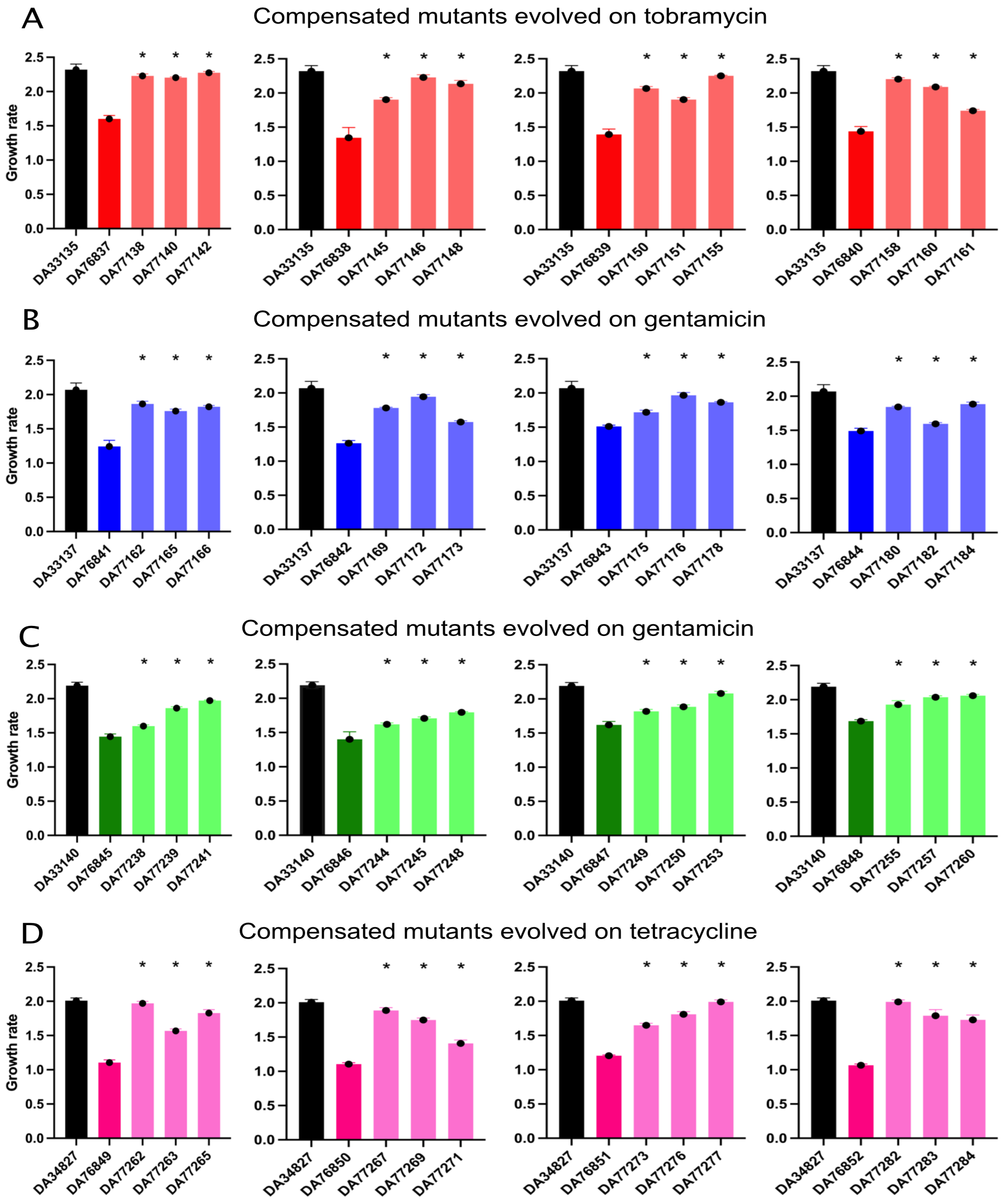

Supplementary Fig. 3. Loss dynamics of the resistant mutants isolated at 24X MIC during 150 generations of growth in absence of antibiotics. (A) *E. coli* isolated tobramycin (B) *E. coli* isolated on gentamicin. (C) *K. pneumoniae* isolated on gentamicin. (D) *S.* Typhimurium isolated on tetracycline.

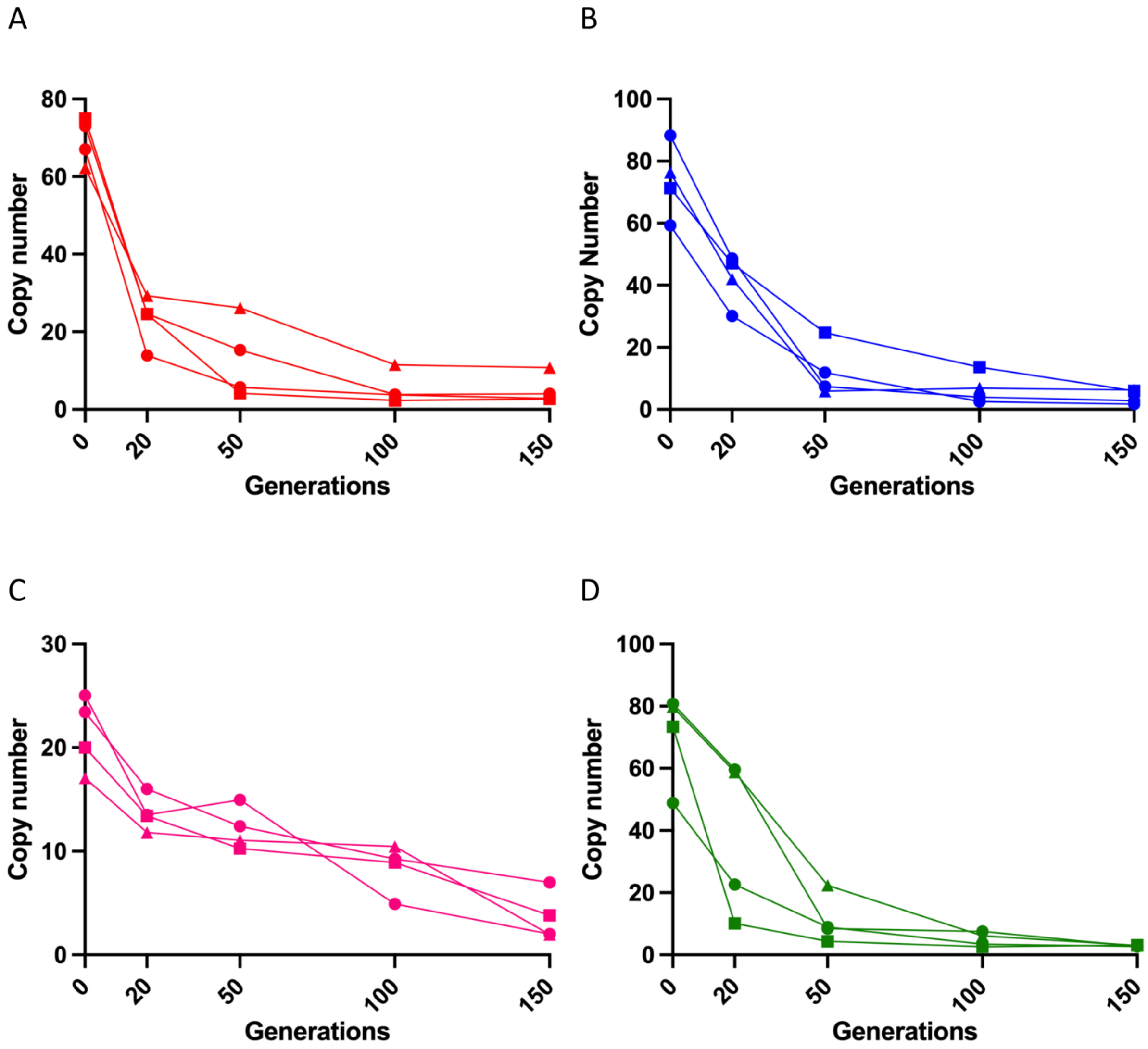

Supplementary Fig. 4. Determination of recombination rates and fitness costs of gene amplifications using homologous recombination model. (A) Loss dynamics of *E. coli* evolved on tobramycin (B) Loss dynamics of *E. coli* evolved on gentamicin. (C) Loss dynamics of *K. pneumoniae* evolved on gentamicin. (D) Loss dynamics of *S.* Typhimurium evolved on tetracycline. The dotted colored lines show experimentally measured copy numbers as a function of generations of growth. The darker shade represents the high copy number mutants isolated at 24X MIC and the lighter shades represent its 3 independent lineages for compensatory evolution experiment. The grey line shows the curve fitted to the experimental data using parameter values k_{rec} and s obtained from the model. The goodness-of-fit was assessed using R-squared in regression analysis between the experimental data and the corresponding values obtained from the recombination model.

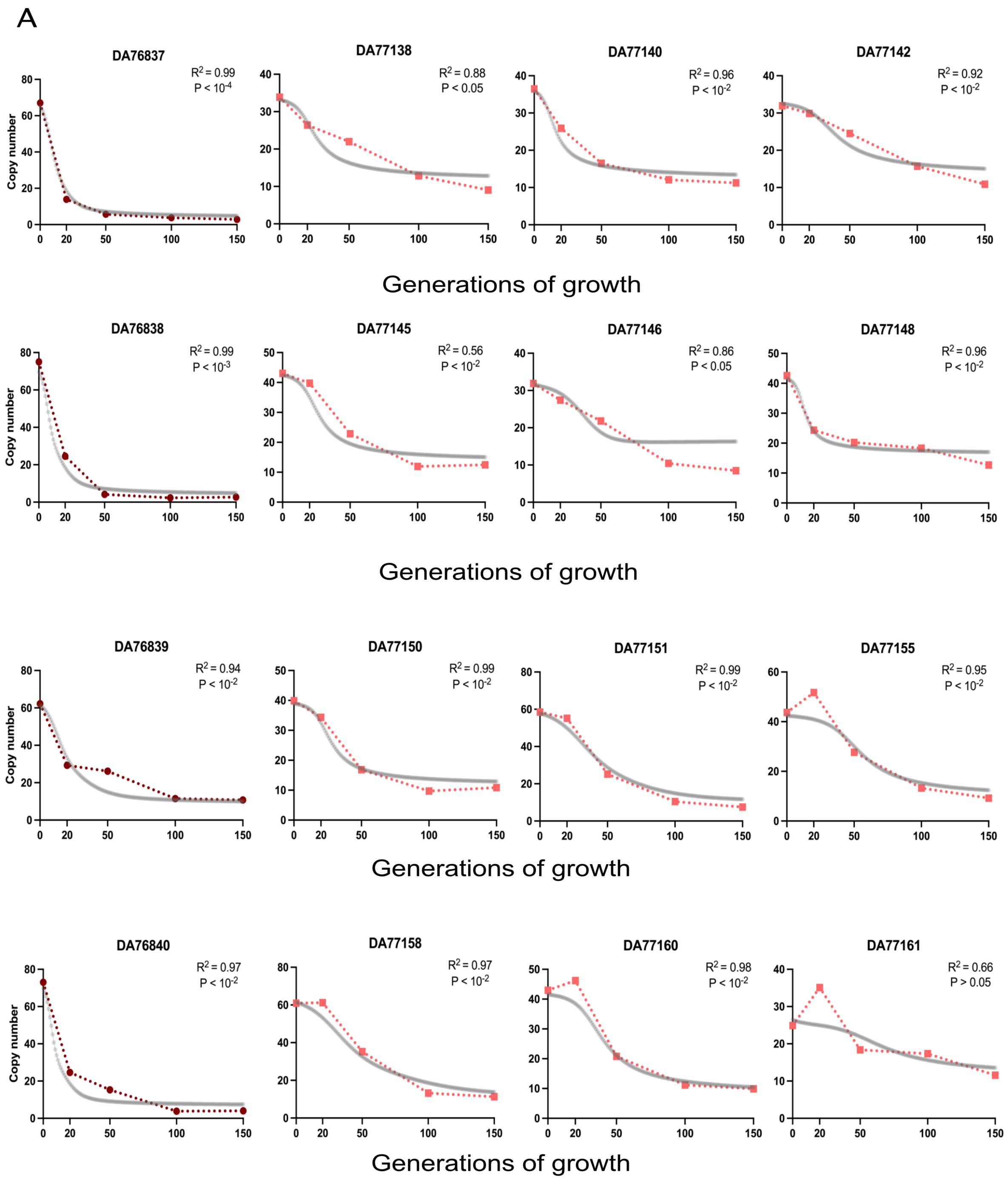

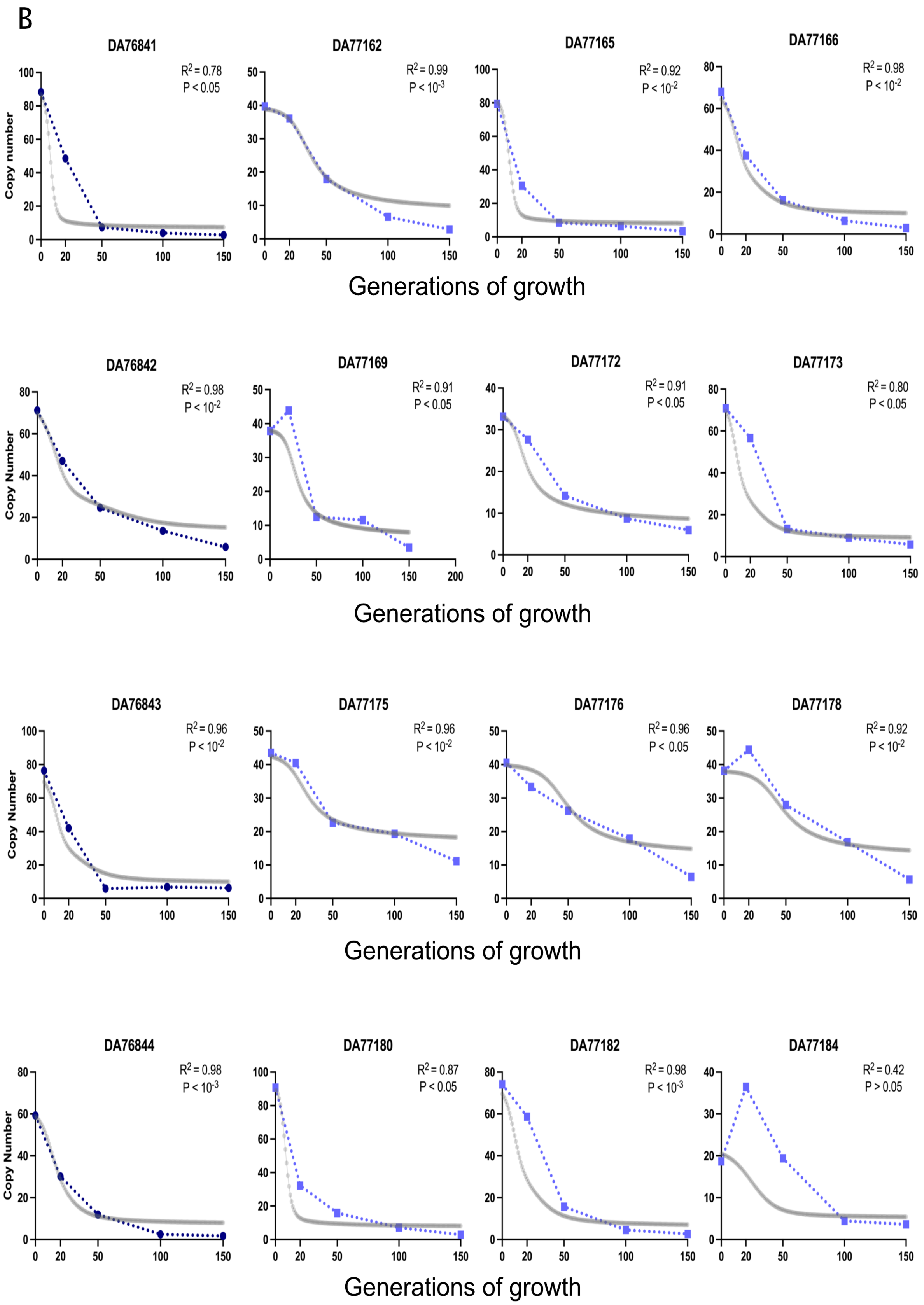

B
DA76841
DA77162
DA77165
DA77166
R2 = 0.78 P < 0.05
R2 = 0.99 P < 10-3
R2 = 0.92 P < 10-2
R2 = 0.98 P < 10-2
Copy number
Generations of growth
DA76842
DA77169
DA77172
DA77173
R2 = 0.98 P < 10-2
R2 = 0.91 P < 0.05
R2 = 0.91 P < 0.05
R2 = 0.80 P < 0.05
Copy Number
Generations of growth
DA76843
DA77175
DA77176
DA77178
R2 = 0.96 P < 10-2
R2 = 0.96 P < 10-2
R2 = 0.96 P < 0.05
R2 = 0.92 P < 10-2
Copy Number
Generations of growth
DA76844
DA77180
DA77182
DA77184
R2 = 0.98 P < 10-3
R2 = 0.87 P < 0.05
R2 = 0.98 P < 10-3
R2 = 0.42 P > 0.05
Copy Number
Generations of growth

C

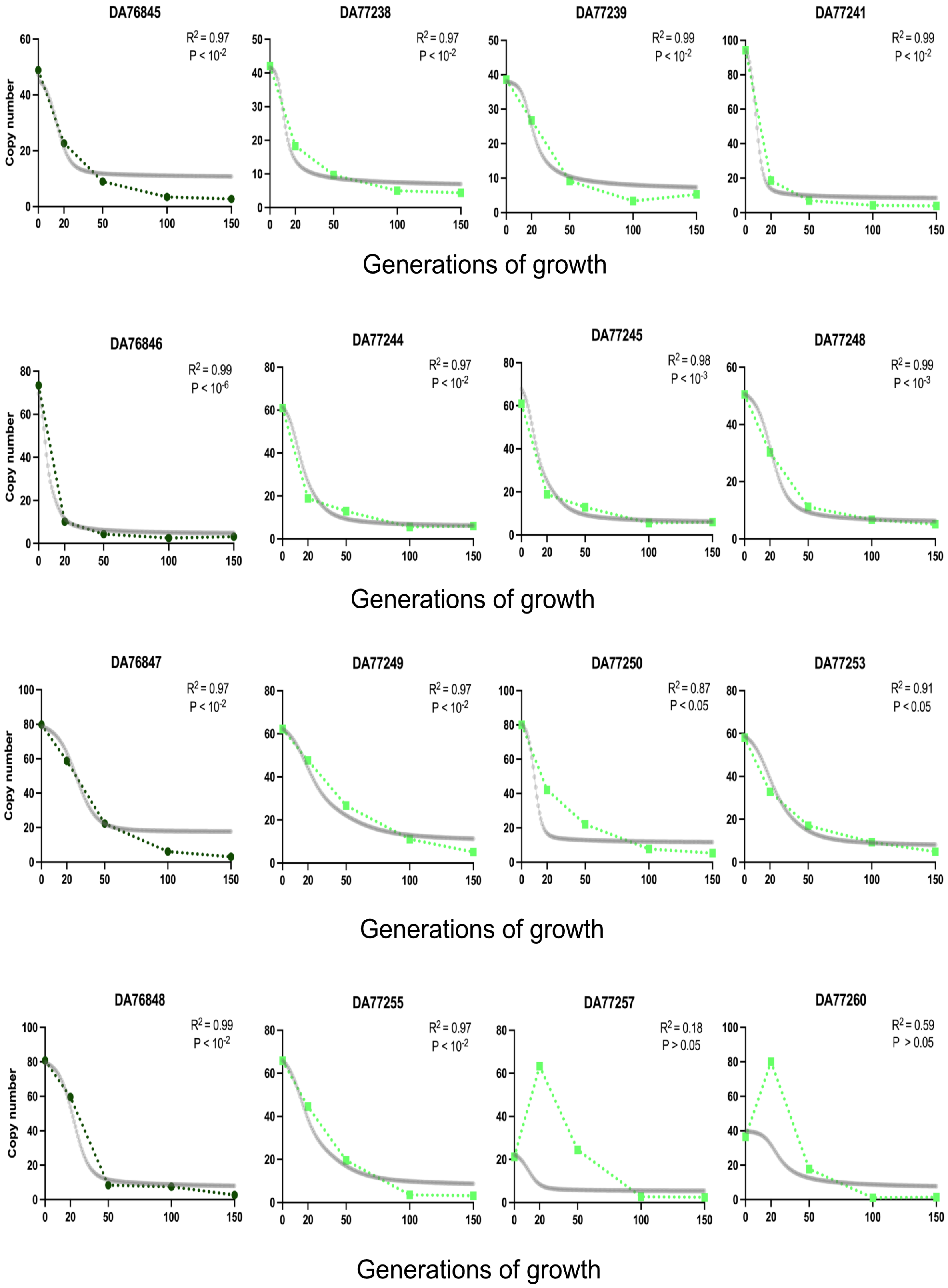
DA76845
R2 = 0.97
P < 10-2
DA77238
R2 = 0.97
P < 10-2
DA77239
R2 = 0.99
P < 10-2
DA77241
R2 = 0.99
P < 10-2
Copy number
Generations of growth
DA76846
R2 = 0.99
P < 10-6
DA77244
R2 = 0.97
P < 10-2
DA77245
R2 = 0.98
P < 10-3
DA77248
R2 = 0.99
P < 10-3
Copy number
Generations of growth
DA76847
R2 = 0.97
P < 10-2
DA77249
R2 = 0.97
P < 10-2
DA77250
R2 = 0.87
P < 0.05
DA77253
R2 = 0.91
P < 0.05
Copy number
Generations of growth
DA76848
R2 = 0.99
P < 10-2
DA77255
R2 = 0.97
P < 10-2
DA77257
R2 = 0.18
P > 0.05
DA77260
R2 = 0.59
P > 0.05
Copy number
Generations of growth

D

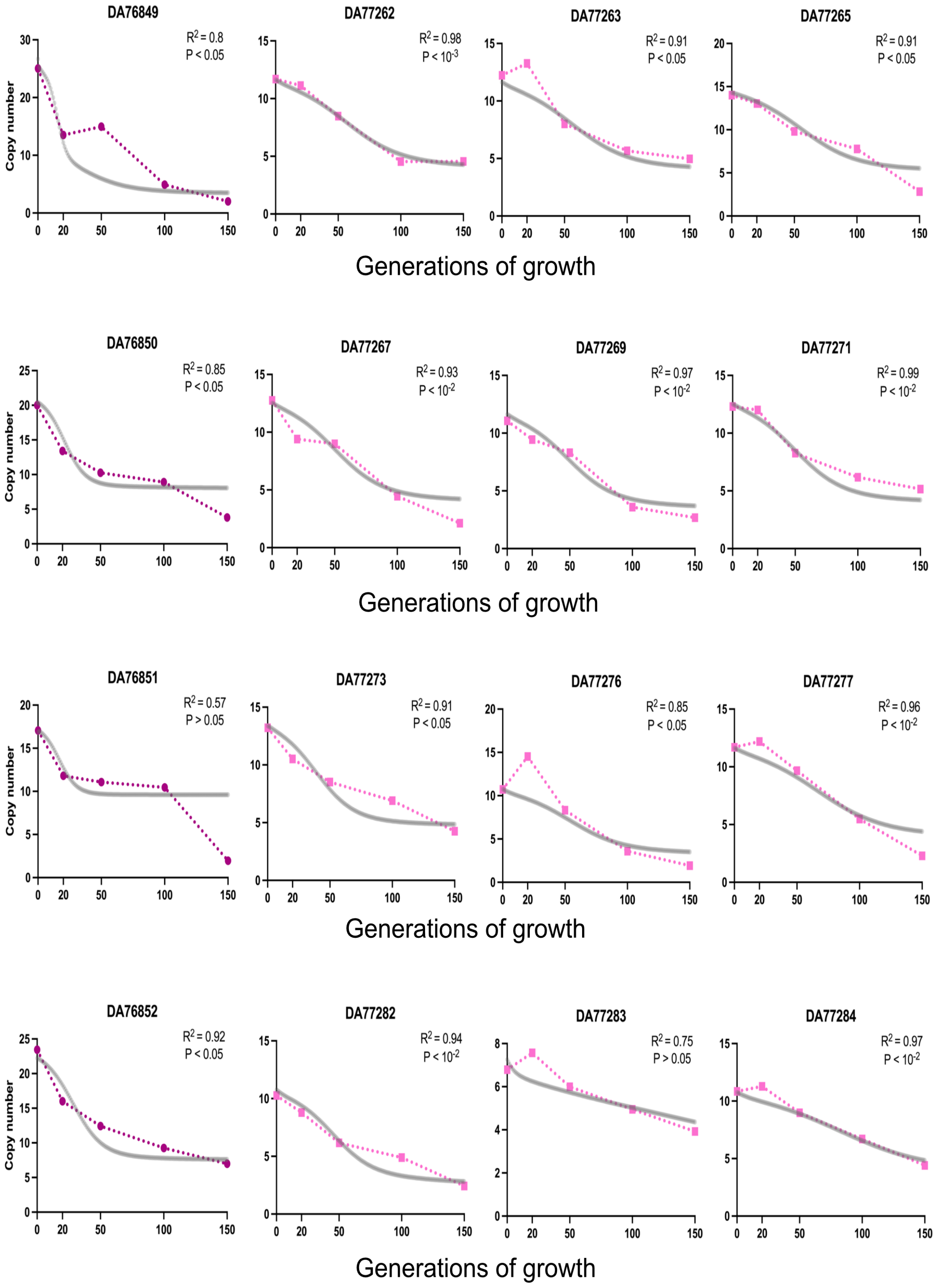

DA76849
$R^2 = 0.8$
$P < 0.05$
DA77262
$R^2 = 0.98$
$P < 10^{-3}$
DA77263
$R^2 = 0.91$
$P < 0.05$
DA77265
$R^2 = 0.91$
$P < 0.05$
Copy number
Generations of growth
DA76850
$R^2 = 0.85$
$P < 0.05$
DA77267
$R^2 = 0.93$
$P < 10^{-2}$
DA77269
$R^2 = 0.97$
$P < 10^{-2}$
DA77271
$R^2 = 0.99$
$P < 10^{-2}$
Copy number
Generations of growth
DA76851
$R^2 = 0.57$
$P > 0.05$
DA77273
$R^2 = 0.91$
$P < 0.05$
DA77276
$R^2 = 0.85$
$P < 0.05$
DA77277
$R^2 = 0.96$
$P < 10^{-2}$
Copy number
Generations of growth
DA76852
$R^2 = 0.92$
$P < 0.05$
DA77282
$R^2 = 0.94$
$P < 10^{-2}$
DA77283
$R^2 = 0.75$
$P > 0.05$
DA77284
$R^2 = 0.97$
$P < 10^{-2}$
Copy number
Generations of growth

Supplementary Table 1. MIC of the resistant mutants isolated at different antibiotic concentrations.

	Antibiotic	Parental strains	1X MIC	4X MIC	16X MIC	24X MIC
DA33135 All lineages	Tobramycin	3 mg/l	3 mg/l	12 mg/l	64 mg/l	> 256 mg/l
DA33137 All lineages	Gentamicin	16 mg/l	16 mg/l	> 256 mg/l	> 256 mg/l	> 256 mg/l
DA33140 All lineages	Gentamicin	24 mg/l	24 mg/l	> 256 mg/l	> 256 mg/l	> 256 mg/l
DA34827 All lineages	Tetracycline	12 mg/l	12 mg/l	> 256 mg/l	> 256 mg/l	> 256 mg/l

Supplementary Table 2. MIC of the compensated mutants isolated at after 100 generations of compensatory evolution on 24X MIC.

Strain number	Species	Antibiotic	MIC (mg/L)
DA77138	*E. coli*	Tobramycin	> 256
DA77140	*E. coli*	Tobramycin	> 256
DA77142	*E. coli*	Tobramycin	> 256
DA77145	*E. coli*	Tobramycin	> 256
DA77146	*E. coli*	Tobramycin	> 256
DA77148	*E. coli*	Tobramycin	> 256
DA77150	*E. coli*	Tobramycin	> 256
DA77151	*E. coli*	Tobramycin	> 256
DA77155	*E. coli*	Tobramycin	> 256
DA77158	*E. coli*	Tobramycin	> 256
DA77160	*E. coli*	Tobramycin	> 256
DA77161	*E. coli*	Tobramycin	> 256
DA77162	*E. coli*	Gentamicin	> 256
DA77165	*E. coli*	Gentamicin	> 256
DA77166	*E. coli*	Gentamicin	> 256
DA77169	*E. coli*	Gentamicin	> 256
DA77172	*E. coli*	Gentamicin	> 256
DA77173	*E. coli*	Gentamicin	> 256
DA77175	*E. coli*	Gentamicin	> 256
DA77176	*E. coli*	Gentamicin	> 256
DA77178	*E. coli*	Gentamicin	> 256
DA77180	*E. coli*	Gentamicin	> 256
DA77182	*E. coli*	Gentamicin	> 256
DA77184	*E. coli*	Gentamicin	> 256

DA77238	*K. pneumoniae*	Gentamicin	> 256
DA77239	*K. pneumoniae*	Gentamicin	> 256
DA77241	*K. pneumoniae*	Gentamicin	> 256
DA77244	*K. pneumoniae*	Gentamicin	> 256
DA77245	*K. pneumoniae*	Gentamicin	> 256
DA77248	*K. pneumoniae*	Gentamicin	> 256
DA77249	*K. pneumoniae*	Gentamicin	> 256
DA77250	*K. pneumoniae*	Gentamicin	> 256
DA77253	*K. pneumoniae*	Gentamicin	> 256
DA77255	*K. pneumoniae*	Gentamicin	> 256
DA77257	*K. pneumoniae*	Gentamicin	> 256
DA77260	*K. pneumoniae*	Gentamicin	> 256
DA77262	*S.* Typhimurium	Tetracycline	> 256
DA77263	*S.* Typhimurium	Tetracycline	> 256
DA77265	*S.* Typhimurium	Tetracycline	> 256
DA77267	*S.* Typhimurium	Tetracycline	> 256
DA77269	*S.* Typhimurium	Tetracycline	> 256
DA77271	*S.* Typhimurium	Tetracycline	> 256
DA77273	*S.* Typhimurium	Tetracycline	> 256
DA77276	*S.* Typhimurium	Tetracycline	> 256
DA77277	*S.* Typhimurium	Tetracycline	> 256
DA77282	*S.* Typhimurium	Tetracycline	> 256
DA77283	*S.* Typhimurium	Tetracycline	> 256
DA77284	*S.* Typhimurium	Tetracycline	> 256

Supplementary Table 3. Identified mutations in the sequenced strains

**DA33135** *E. coli*	**Strain number**	**CDP-glycerol phosphotransferase family protein**	**Structural variants**
Compensated mutants	DA77138		c.*qorB*::1249340^1249539::Insertion
	DA77140		c.*lrhA*::1249340..1249539::Replacement<br>c.*hyxA*::3408804^3408813::Insertion
	DA77144	c.TCT>TTT<br>S147F	c.*hyxA*::3408804^3408813::Insertion<br>c.*fimA/fimE*::3813219..3813532::Deletion<br>c.*fimE*::3813904^3813913::Insertion

**DA33135** *E. coli*	**Strain number**	***yehB***	***katE/cedA***	**Structural variants**
Compensated mutants	DA77145			c.DLJ63_RS28170::16525^16534::Insertion
	DA77146			c. *kup*::45555672^4555681::Insertion
	DA77148	c.CAA>CCA<br>Q50P	c.1890740::C>A	c.DLJ63_RS00125::19177^19186::Insertion

**DA33135** *E. coli*	**Strain number**	***rluD***	***lrhA***	***pgsA***	***traU***	***fimA/fimE***	**Structural variants**
Compensated mutants	DA77150	c.875813:: Δ6bp					c. *gspK*::21883^21895::Insertion
	DA77151		c. 1248795::Δ1563bp				c. *yiaO*::4744083^4744092::Insertion<br>c. *gspK*::21883^21895::Insertion
	DA77155			c.CTA>CA A<br>L108Q	c. 676Del<br>Q221 fs	c.3813536::T> C	c.DLJ63_RS28170::16525^16434::Insertion

**DA33135** *E. coli*	**Strain number**	***nuoG***	***lrhA***	***ybbY***	**Structural variants**
Compensated mutants	DA77158				c. Uxa family hydrolase::3011692^3011702::Insertion<br>c. *fimA/fimE*::3813219..3813532::Deletion<br>c. *fimE*::3813709^3813718::Insertion<br>c. *bglB*::4585803^4585812::Insertion
	DA77160	c.TTT>GTT<br>F33V			c. *bglB*::4585803^4585812::Insertion
	DA77161		c.GTT>GAT<br>V225D	c.CTG>CTT<br>L398L	c. *bglB*::4585803^4585812::Insertion<br>c. *pitA*::4861030^4861039::Insertion

**DA33137** *E. coli*	**Strain number**	***cyoB***	***fimE***	**MGMT family protein**	**Hypothetical protein**	**Structural variants**
Compensated mutants	DA77162	c.CGT>GGT R71G	c.GAA>GCA E184A			
	DA77165					c. *hns/tdk*::4907245^4907255::Insertion
	DA77166			c.CCC>CCG P36P	c.GGT>GGG G46G	

**DA33137** *E. coli*	**Strain number**	***cpxR***	**DLJ63_RS00070**	**Structural variants**
Compensated mutants	DA77169			c. 162734.. 174199::Δ11.4 kbp Deletion
	DA77172	c.CTG>CA G L20Q		c. 162734.. 174205::Δ11.4 kbp Deletion
	DA77173		c.CCA>CTA P1351L	c. 162735.. 174189::Δ11.4kbp Deletion c.*lrhA*::4262946..4263145::Replacement

**DA33137** *E. coli*	**Strain number**	***tkt/cmtB***	***cpxA***
Compensated mutants	DA77175	c.3573588::A>G	
	DA77176		c.GAT>GAG D245E
	DA77178		

**DA33137** *E. coli*	**Strain number**	***rsmF***	**CDP-glycerol glycerophosphate family protein**	**Structural variants**
Parent 4	DA76844			
Compensated mutants	DA77180			
	DA77182	c.TTG>TAG L106*	c.3423953::Δ1bp	c.*lrhA*::4262946..4263145::Replacement
	DA77184	c.TTG>TAG L106*	c.CCC>CCG P36P	c. *fimA/fimE*::1888698..1889001::Deletion

**DA33140** *K. pneumoniae*	**Strain number**	***pta***	**Hypothetical protein**	***cpxA***	***nuoA***	**DLJ63_RS22460/ DLJ63_RS22470**	**Structural variants**
Compensated mutants	DA77238	c.CGT>GGT R71G	c.GAA>GCA E184A	c.2173373::Δ3bp			c. *wcaJ*::258042^258052::Insertion
	DA77239				c. 478508::Δ2bp	c. 4551847::+1bp	c. *hns/tdk*::4907245^4907255::Insertion
	DA77241				c. 478520::Δ2bp		p. 2318^7020::Δ4702bp

**DA33140** *K. pneumoniae*	**Strain number**	***wcaJ***	***lpxM***	***arcB***
Compensated mutants	DA77244	c.AAA>TAA K47*	c.5423233::Δ2bp	
	DA77245	c.CAA>TAA Q21*		c.GAG>GTG E78V
	DA77248			

**DA33140** *K. pneumoniae*	**Strain number**	***cyoE***	**DLJ83_RS15700**	***iroE***	**DLJ83_RS29515/ DLJ83_RS27805**	**DLJ83_RS01335**	**Structural variants**
Compensated mutants	DA77249	c.CGT>GGT R71G	c.GAA>GCA E184A	c.TGG>TGA W280*	p. 7019::T>C p. 7026::T>G	c. 273058^283059::+129bp	c. *wcaJ*::258042^258052::Insertion
	DA77250						c. 258586^258596::Insertion
	DA77253						

**DA33140** *K. pneumoniae*	**Strain number**	***rpoA***	**DLJ83_RS19860/*ghrA***	**Structural variants**
Compensated mutants	DA77255	c.GGT>GGG G3G	c. 4044877::G>C	
	DA77257		c. 4044877::G>C	c. *cyoA*::3311661^3311672::Insertion
	DA77260		c. 4044877::G>C	c. *wcaJ*::258585^3311672::Insertion

**DA34827** *S.* Typhimurium	**Strain number**	**TetR/AcrR family TR/RamA family antibiotic efflux TR**	***rcsB***	**Structural variants**
Compensated mutants	DA77262	c.3122748::C>T		c.4072404::Δ24 kbp
	DA77263	c.3122748::C>T		
	DA77265	c.3122748::C>T	c.CCG>CTG P60L	c. *rcnA*::744963^744964::+6bp

**DA34827** *S.* Typhimurium	**Strain number**	**TetR/AcrR family TR/RamA family antibioticefflux TR**	**TetR/AcrR family TR**	***rcsB***	**MerR family TR**	**Structural variants**
Compensated mutants	DA77267	c.3122748::C>T	c.CTG>CCG L179P			c.*YmgB*/AriR family protein::3790115::Δ143 bp
	DA77269		c.CTG>CCG L179P	c.CCG>CTG P60L		
	DA77271		c.CTG>CCG L179P		c.GAA>AAA E85K	

**DA34827** *S.* Typhimurium	**Strain number**	**TetR/AcrR family TR**	***rcsB***	***rho***	***yodD*/DLJ69_RS23485**	**Structural variants**
Compensated mutants	DA77273	c.TTT>CTT F45L				c.3789474::Δ7.5 kbp
	DA77276	c.TTT>CTT F45L	c.GGT>AGT G111S			
	DA77277	c.TTT>CTT F45L		c.GGT>GAT G99D	c.4556176::A>C	

**DA34827** *S.* Typhimurium	**Strain number**	**TetR/AcrR family TR**	***rpoS***	**Structural variants**
Compensated mutants	DA77282	c.3122451::Δ1 bp		
	DA77283	c.3122451::Δ1 bp		c. 4070488::Δ64 kbp
	DA77284	c.3122451::Δ1 bp	c.628031::+2 bp	

Supplementary Table 4. Resistance levels in reconstructed mutants

**Species**	**Mutation**	**Antibiotic**	**MIC in WT**	**MIC in mutant**	**Relative growth rate in mutant compared to WT**
E. coli	*nuoG::F33V*	Tobramycin	0.1 ± 0.02	0.15 ± 0.04	0.98 ± 0.01
E. coli	*cyoB::R71G*	Gentamicin	0.15 ± 0.4	0.625 ± 0.18	0.98 ± 0.01
S. Typhimurium	*tet(R)/acrR::L179P*	Tetracycline	0.5 ± 0	1.5 ± 0	0.96 ± 0.01
S. Typhimurium	*rcsB::P60L*	Tetracycline	0.5 ± 0	1 ± 0	0.96 ± 0.02

Supplementary Table 5. Recombination rates (k_{rec}) and fitness costs (*s*) values for resistant and compensated mutants

**Strain**	**Antibiotic**	**Description**	**Recombination rate (k_{rec})**	**Cost of amplification (s)**
DA76837	Tobramycin	Resistant mutant isolated at 24X MIC	0.0001	-0.009
DA77138	Tobramycin	Compensated mutant after 100 generations	0.0015	-0.014
DA77140	Tobramycin	Compensated mutant after 100 generations	0.0024	-0.019
DA77142	Tobramycin	Compensated mutant after 100 generations	0.0015	0.01
DA76838	Tobramycin	Resistant mutant isolated at 24X MIC	0.0001	-0.009
DA77145	Tobramycin	Compensated mutant after 100 generations	0.0014	-0.007
DA77146	Tobramycin	Compensated mutant after 100 generations	0.005	-0.011
DA77148	Tobramycin	Compensated mutant after 100 generations	0.004	-0.014
DA76839	Tobramycin	Resistant mutant isolated at 24X MIC	0.0002	-0.006
DA77150	Tobramycin	Compensated mutant after 100 generations	0.0008	-0.009
DA77151	Tobramycin	Compensated mutant after 100 generations	0.0001	-0.003
DA77155	Tobramycin	Compensated mutant after 100 generations	0.0001	-0.003
DA76840	Tobramycin	Resistant mutant isolated at 24X MIC	0.0002	-0.01
DA77158	Tobramycin	Compensated mutant after 100 generations	0.0001	-0.0025
DA77160	Tobramycin	Compensated mutant after 100 generations	0.0001	-0.005
DA77161	Tobramycin	Compensated mutant after 100 generations	0.0012	-0.011
DA76841	Gentamicin	Resistant mutant isolated at 24X MIC	0.0001	-0.008
DA77162	Gentamicin	Compensated mutant after 100 generations	0.00015	-0.006
DA77165	Gentamicin	Compensated mutant after 100 generations	0.0001	-0.007
DA77166	Gentamicin	Compensated mutant after 100 generations	0.0002	-0.006
DA76842	Gentamicin	Resistant mutant isolated at 24X MIC	0.0003	-0.004
DA77169	Gentamicin	Compensated mutant after 100 generations	0.0001	-0.008
DA77172	Gentamicin	Compensated mutant after 100 generations	0.0003	-0.015
DA77173	Gentamicin	Compensated mutant after 100 generations	0.0002	-0.007
DA76843	Gentamicin	Resistant mutant isolated at 24X MIC	0.0002	-0.006
DA77175	Gentamicin	Compensated mutant after 100 generations	0.0012	-0.006
DA77176	Gentamicin	Compensated mutant after 100 generations	0.0004	-0.004
DA77178	Gentamicin	Compensated mutant after 100 generations	0.0005	-0.005
DA76844	Gentamicin	Resistant mutant isolated at 24X MIC	0.00015	-0.007
DA77180	Gentamicin	Compensated mutant after 100 generations	0.0001	-0.007

DA77182	Gentamicin	Compensated mutant after 100 generations	0.0001	-0.006
DA77184	Gentamicin	Compensated mutant after 100 generations	0.001	-0.008
DA76845	Gentamicin	Resistant mutant isolated at 24X MIC	0.0008	-0.008
DA77238	Gentamicin	Compensated mutant after 100 generations	0.00035	-0.018
DA77239	Gentamicin	Compensated mutant after 100 generations	0.0002	-0.011
DA77241	Gentamicin	Compensated mutant after 100 generations	0.0001	-0.0065
DA76846	Gentamicin	Resistant mutant isolated at 24X MIC	0.00015	-0.013
DA77244	Gentamicin	Compensated mutant after 100 generations	0.0001	-0.007
DA77245	Gentamicin	Compensated mutant after 100 generations	0.0001	-0.007
DA77248	Gentamicin	Compensated mutant after 100 generations	0.0001	-0.007
DA76847	Gentamicin	Resistant mutant isolated at 24X MIC	0.0002	-0.003
DA77249	Gentamicin	Compensated mutant after 100 generations	0.00015	-0.004
DA77250	Gentamicin	Compensated mutant after 100 generations	0.0002	-0.007
DA77253	Gentamicin	Compensated mutant after 100 generations	0.0001	-0.005
DA76848	Gentamicin	Resistant mutant isolated at 24X MIC	0.000035	-0.003
DA77255	Gentamicin	Compensated mutant after 100 generations	0.0001	-0.0045
DA77257	Gentamicin	Compensated mutant after 100 generations	0.002	-0.015
DA77260	Gentamicin	Compensated mutant after 100 generations	0.0001	-0.008
DA76849	Tetracycline	Resistant mutant isolated at 24X MIC	0.0004	-0.021
DA77262	Tetracycline	Compensated mutant after 100 generations	0.001	-0.012
DA77263	Tetracycline	Compensated mutant after 100 generations	0.001	-0.012
DA77265	Tetracycline	Compensated mutant after 100 generations	0.001	-0.007
DA76850	Tetracycline	Resistant mutant isolated at 24X MIC	0.0037	-0.01
DA77267	Tetracycline	Compensated mutant after 100 generations	0.0009	-0.011
DA77269	Tetracycline	Compensated mutant after 100 generations	0.0009	-0.013
DA77271	Tetracycline	Compensated mutant after 100 generations	0.0009	-0.011
DA76851	Tetracycline	Resistant mutant isolated at 24X MIC	0.02	-0.025
DA77273	Tetracycline	Compensated mutant after 100 generations	0.0014	-0.013
DA77276	Tetracycline	Compensated mutant after 100 generations	0.0009	-0.014
DA77277	Tetracycline	Compensated mutant after 100 generations	0.0008	-0.01
DA76852	Tetracycline	Resistant mutant isolated at 24X MIC	0.0017	-0.006
DA77282	Tetracycline	Compensated mutant after 100 generations	0.0007	-0.015
DA77282	Tetracycline	Compensated mutant after 100 generations	0.0007	-0.015
DA77284	Tetracycline	Compensated mutant after 100 generations	0.0008	-0.01

Supplementary Table 6. List of strains used in the study

Strain Number	Species	Reference/ Source	Comment
DA33135	*E. coli*		Clinical isolate ECO-005
DA33137	*E. coli*		Clinical isolate ECO-033
DA33140	*K. pneumoniae*		Clinical isolate
DA34827	*S.* Typhimurium		Clinical isolate from feces, Sweden
DA76789	*E. coli*	This study	DA33135 grown at 1X MIC tobramycin on agar plate
DA76790	*E. coli*	This study	DA33135 grown at 1X MIC tobramycin on agar plate
DA76791	*E. coli*	This study	DA33135 grown at 1X MIC tobramycin on agar plate
DA76792	*E. coli*	This study	DA33135 grown at 1X MIC tobramycin on agar plate
DA76793	*E. coli*	This study	DA33137 grown at 1X MIC gentamicin on agar plate
DA76794	*E. coli*	This study	DA33137 grown at 1X MIC gentamicin on agar plate
DA76795	*E. coli*	This study	DA33137 grown at 1X MIC gentamicin on agar plate
DA76796	*E. coli*	This study	DA33137 grown at 1X MIC gentamicin on agar plate
DA76797	*K. pneumoniae*	This study	DA33140 grown at 1X MIC gentamicin on agar plate
DA76798	*K. pneumoniae*	This study	DA33140 grown at 1X MIC gentamicin on agar plate
DA76799	*K. pneumoniae*	This study	DA33140 grown at 1X MIC gentamicin on agar plate
DA76800	*K. pneumoniae*	This study	DA33140 grown at 1X MIC gentamicin on agar plate
DA76801	*S.* Typhimurium	This study	DA34827 grown at 1X MIC tetracycline on agar plate
DA76802	*S.* Typhimurium	This study	DA34827 grown at 1X MIC tetracycline on agar plate
DA76803	*S.* Typhimurium	This study	DA34827 grown at 1X MIC tetracycline on agar plate
DA76804	*S.* Typhimurium	This study	DA34827 grown at 1X MIC tetracycline on agar plate
DA76805	*E. coli*	This study	DA76789 evolved at 4X MIC tobramycin on agar plate
DA76806	*E. coli*	This study	DA76790 evolved at 4X MIC tobramycin on agar plate
DA76807	*E. coli*	This study	DA76791 evolved at 4X MIC tobramycin on agar plate
DA76808	*E. coli*	This study	DA76792 evolved at 4X MIC tobramycin on agar plate
DA76809	*E. coli*	This study	DA76793 evolved at 4X MIC gentamicin on agar plate
DA76810	*E. coli*	This study	DA76794 evolved at 4X MIC gentamicin on agar plate
DA76811	*E. coli*	This study	DA76795 evolved at 4X MIC gentamicin on agar plate
DA76812	*E. coli*	This study	DA76796 evolved at 4X MIC gentamicin on agar plate
DA76813	*K. pneumoniae*	This study	DA76797 evolved at 4X MIC gentamicin on agar plate
DA76814	*K. pneumoniae*	This study	DA76798 evolved at 4X MIC gentamicin on agar plate
DA76815	*K. pneumoniae*	This study	DA76799 evolved at 4X MIC gentamicin on agar plate
DA76816	*K. pneumoniae*	This study	DA76800 evolved at 4X MIC gentamicin on agar plate
DA76817	*S.* Typhimurium	This study	DA76801 evolved at 4X MIC tetracycline on agar plate
DA76818	*S.* Typhimurium	This study	DA76802 evolved at 4X MIC tetracycline on agar plate
DA76819	*S.* Typhimurium	This study	DA76803 evolved at 4X MIC tetracycline on agar plate
DA76820	*S.* Typhimurium	This study	DA76804 evolved at 4X MIC tetracycline on agar plate
DA76821	*E. coli*	This study	DA76805 evolved at 16X MIC tobramycin on agar plate
DA76822	*E. coli*	This study	DA76806 evolved at 16X MIC tobramycin on agar plate
DA76823	*E. coli*	This study	DA76807 evolved at 16X MIC tobramycin on agar plate
DA76824	*E. coli*	This study	DA76808 evolved at 16X MIC tobramycin on agar plate
DA76825	*E. coli*	This study	DA76809 evolved at 16X MIC gentamicin on agar plate
DA76826	*E. coli*	This study	DA76810 evolved at 16X MIC gentamicin on agar plate
DA76827	*E. coli*	This study	DA76811 evolved at 16X MIC gentamicin on agar plate
DA76828	*E. coli*	This study	DA76812 evolved at 16X MIC gentamicin on agar plate
DA76829	*K. pneumoniae*	This study	DA76813 evolved at 16X MIC gentamicin on agar plate
DA76830	*K. pneumoniae*	This study	DA76814 evolved at 16X MIC gentamicin on agar plate
DA76831	*K. pneumoniae*	This study	DA76815 evolved at 16X MIC gentamicin on agar plate
DA76832	*K. pneumoniae*	This study	DA76816 evolved at 16X MIC gentamicin on agar plate
DA76833	*S.* Typhimurium	This study	DA76817 evolved at 16X MIC tetracycline on agar plate
DA76834	*S.* Typhimurium	This study	DA76818 evolved at 16X MIC tetracycline on agar plate
DA76835	*S.* Typhimurium	This study	DA76819 evolved at 16X MIC tetracycline on agar plate
DA76836	*S.* Typhimurium	This study	DA76820 evolved at 16X MIC tetracycline on agar plate
DA76837	*E. coli*	This study	DA76821 evolved at 24X MIC tobramycin on agar plate
DA76838	*E. coli*	This study	DA76822 evolved at 24X MIC tobramycin on agar plate
DA76839	*E. coli*	This study	DA76823 evolved at 24X MIC tobramycin on agar plate
DA76840	*E. coli*	This study	DA76824 evolved at 24X MIC tobramycin on agar plate
DA76841	*E. coli*	This study	DA76825 evolved at 24X MIC gentamicin on agar plate
DA76842	*E. coli*	This study	DA76826 evolved at 24X MIC gentamicin on agar plate
DA76843	*E. coli*	This study	DA76827 evolved at 24X MIC gentamicin on agar plate
DA76844	*E. coli*	This study	DA76828 evolved at 24X MIC gentamicin on agar plate

DA76845	*K. pneumoniae*	This study	DA76829 evolved at 24X MIC gentamicin on agar plate
DA76846	*K. pneumoniae*	This study	DA76830 evolved at 24X MIC gentamicin on agar plate
DA76847	*K. pneumoniae*	This study	DA76831 evolved at 24X MIC gentamicin on agar plate
DA76848	*K. pneumoniae*	This study	DA76832 evolved at 24X MIC gentamicin on agar plate
DA76849	*S.* Typhimurium	This study	DA76833 evolved at 24X MIC tetracycline on agar plate
DA76850	*S.* Typhimurium	This study	DA76834 evolved at 24X MIC tetracycline on agar plate
DA76851	*S.* Typhimurium	This study	DA76835 evolved at 24X MIC tetracycline on agar plate
DA76852	*S.* Typhimurium	This study	DA76836 evolved at 24X MIC tetracycline on agar plate
DA77138	*E. coli*	This study	Compensatory evolution of DA76837 on 24X MIC tobramycin (100 generations)
DA77140	*E. coli*	This study	Compensatory evolution of DA76837 on 24X MIC tobramycin (100 generations)
DA77142	*E. coli*	This study	Compensatory evolution of DA76837 on 24X MIC tobramycin (100 generations)
DA77145	*E. coli*	This study	Compensatory evolution of DA76838 on 24X MIC tobramycin (100 generations)
DA77146	*E. coli*	This study	Compensatory evolution of DA76838 on 24X MIC tobramycin (100 generations)
DA77148	*E. coli*	This study	Compensatory evolution of DA76838 on 24X MIC tobramycin (100 generations)
DA77150	*E. coli*	This study	Compensatory evolution of DA76839 on 24X MIC tobramycin (100 generations)
DA77151	*E. coli*	This study	Compensatory evolution of DA76839 on 24X MIC tobramycin (100 generations)
DA77155	*E. coli*	This study	Compensatory evolution of DA76839 on 24X MIC tobramycin (100 generations)
DA77158	*E. coli*	This study	Compensatory evolution of DA76840 on 24X MIC tobramycin (100 generations)
DA77160	*E. coli*	This study	Compensatory evolution of DA76840 on 24X MIC tobramycin (100 generations)
DA77161	*E. coli*	This study	Compensatory evolution of DA76840 on 24X MIC tobramycin (100 generations)
DA77162	*E. coli*	This study	Compensatory evolution of DA76841 on 24X MIC gentamicin (100 generations)
DA77165	*E. coli*	This study	Compensatory evolution of DA76841 on 24X MIC gentamicin (100 generations)
DA77166	*E. coli*	This study	Compensatory evolution of DA76841 on 24X MIC gentamicin (100 generations)
DA77169	*E. coli*	This study	Compensatory evolution of DA76842 on 24X MIC gentamicin (100 generations)
DA77172	*E. coli*	This study	Compensatory evolution of DA76842 on 24X MIC gentamicin (100 generations)
DA77173	*E. coli*	This study	Compensatory evolution of DA76842 on 24X MIC gentamicin (100 generations)
DA77175	*E. coli*	This study	Compensatory evolution of DA76843 on 24X MIC gentamicin (100 generations)
DA77176	*E. coli*	This study	Compensatory evolution of DA76843 on 24X MIC gentamicin (100 generations)
DA77178	*E. coli*	This study	Compensatory evolution of DA76843 on 24X MIC gentamicin (100 generations)
DA77180	*E. coli*	This study	Compensatory evolution of DA76844 on 24X MIC gentamicin (100 generations)
DA77182	*E. coli*	This study	Compensatory evolution of DA76844 on 24X MIC gentamicin (100 generations)
DA77184	*E. coli*	This study	Compensatory evolution of DA76844 on 24X MIC gentamicin (100 generations)
DA77238	*K. pneumoniae*	This study	Compensatory evolution of DA76845 on 24X MIC gentamicin (100 generations)
DA77239	*K. pneumoniae*	This study	Compensatory evolution of DA76845 on 24X MIC gentamicin (100 generations)
DA77241	*K. pneumoniae*	This study	Compensatory evolution of DA76845 on 24X MIC gentamicin (100 generations)
DA77244	*K. pneumoniae*	This study	Compensatory evolution of DA76846 on 24X MIC gentamicin (100 generations)
DA77245	*K. pneumoniae*	This study	Compensatory evolution of DA76846 on 24X MIC gentamicin (100 generations)
DA77248	*K. pneumoniae*	This study	Compensatory evolution of DA76846 on 24X MIC gentamicin (100 generations)
DA77249	*K. pneumoniae*	This study	Compensatory evolution of DA76847 on 24X MIC gentamicin (100 generations)
DA77250	*K. pneumoniae*	This study	Compensatory evolution of DA76847 on 24X MIC gentamicin (100 generations)
DA77253	*K. pneumoniae*	This study	Compensatory evolution of DA76847 on 24X MIC gentamicin (100 generations)
DA77255	*K. pneumoniae*	This study	Compensatory evolution of DA76848 on 24X MIC gentamicin (100 generations)
DA77257	*K. pneumoniae*	This study	Compensatory evolution of DA76848 on 24X MIC gentamicin (100 generations)
DA77260	*K. pneumoniae*	This study	Compensatory evolution of DA76848 on 24X MIC gentamicin (100 generations)
DA77262	*S.* Typhimurium	This study	Compensatory evolution of DA76849 on 24X MIC tetracycline (100 generations)
DA77263	*S.* Typhimurium	This study	Compensatory evolution of DA76849 on 24X MIC tetracycline (100 generations)
DA77265	*S.* Typhimurium	This study	Compensatory evolution of DA76849 on 24X MIC tetracycline (100 generations)
DA77267	*S.* Typhimurium	This study	Compensatory evolution of DA76850 on 24X MIC tetracycline (100 generations)
DA77269	*S.* Typhimurium	This study	Compensatory evolution of DA76850 on 24X MIC tetracycline (100 generations)
DA77271	*S.* Typhimurium	This study	Compensatory evolution of DA76850 on 24X MIC tetracycline (100 generations)
DA77273	*S.* Typhimurium	This study	Compensatory evolution of DA76851 on 24X MIC tetracycline (100 generations)
DA77276	*S.* Typhimurium	This study	Compensatory evolution of DA76851 on 24X MIC tetracycline (100 generations)
DA77277	*S.* Typhimurium	This study	Compensatory evolution of DA76851 on 24X MIC tetracycline (100 generations)
DA77282	*S.* Typhimurium	This study	Compensatory evolution of DA76852 on 24X MIC tetracycline (100 generations)
DA77283	*S.* Typhimurium	This study	Compensatory evolution of DA76852 on 24X MIC tetracycline (100 generations)
DA77284	*S.* Typhimurium	This study	Compensatory evolution of DA76852 on 24X MIC tetracycline (100 generations)
DA77764	*E. coli*	This study	Evolution of DA77138 in MH at generation 20
DA77765	*E. coli*	This study	Evolution of DA77140 in MH at generation 20
DA77766	*E. coli*	This study	Evolution of DA77142 in MH at generation 20
DA77767	*E. coli*	This study	Evolution of DA77145 in MH at generation 20
DA77768	*E. coli*	This study	Evolution of DA77146 in MH at generation 20
DA77769	*E. coli*	This study	Evolution of DA77148 in MH at generation 20
DA77770	*E. coli*	This study	Evolution of DA77150 in MH at generation 20
DA77771	*E. coli*	This study	Evolution of DA77151 in MH at generation 20
DA77772	*E. coli*	This study	Evolution of DA77155 in MH at generation 20
DA77773	*E. coli*	This study	Evolution of DA77158 in MH at generation 20
DA77774	*E. coli*	This study	Evolution of DA77160 in MH at generation 20
DA77775	*E. coli*	This study	Evolution of DA77161 in MH at generation 20

DA77776	*E. coli*	This study	Evolution of DA77161 in MH at generation 20
DA77777	*E. coli*	This study	Evolution of DA77165 in MH at generation 20
DA77778	*E. coli*	This study	Evolution of DA77166 in MH at generation 20
DA77779	*E. coli*	This study	Evolution of DA77169 in MH at generation 20
DA77780	*E. coli*	This study	Evolution of DA77172 in MH at generation 20
DA77781	*E. coli*	This study	Evolution of DA77173 in MH at generation 20
DA77782	*E. coli*	This study	Evolution of DA77175 in MH at generation 20
DA77783	*E. coli*	This study	Evolution of DA77176 in MH at generation 20
DA77784	*E. coli*	This study	Evolution of DA77178 in MH at generation 20
DA77785	*E. coli*	This study	Evolution of DA77180 in MH at generation 20
DA77786	*E. coli*	This study	Evolution of DA77182 in MH at generation 20
DA77787	*E. coli*	This study	Evolution of DA77184 in MH at generation 20
DA77788	*K. pneumoniae*	This study	Evolution of DA77238 in MH at generation 20
DA77789	*K. pneumoniae*	This study	Evolution of DA77239 in MH at generation 20
DA77790	*K. pneumoniae*	This study	Evolution of DA77241 in MH at generation 20
DA77791	*K. pneumoniae*	This study	Evolution of DA77244 in MH at generation 20
DA77792	*K. pneumoniae*	This study	Evolution of DA77245 in MH at generation 20
DA77793	*K. pneumoniae*	This study	Evolution of DA77248 in MH at generation 20
DA77794	*K. pneumoniae*	This study	Evolution of DA77249 in MH at generation 20
DA77795	*K. pneumoniae*	This study	Evolution of DA77250 in MH at generation 20
DA77796	*K. pneumoniae*	This study	Evolution of DA77253 in MH at generation 20
DA77797	*K. pneumoniae*	This study	Evolution of DA77255 in MH at generation 20
DA77798	*K. pneumoniae*	This study	Evolution of DA77257 in MH at generation 20
DA77799	*K. pneumoniae*	This study	Evolution of DA77260 in MH at generation 20
DA77800	*S.* Typhimurium	This study	Evolution of DA77262 in MH at generation 20
DA77801	*S.* Typhimurium	This study	Evolution of DA77263 in MH at generation 20
DA77802	*S.* Typhimurium	This study	Evolution of DA77265 in MH at generation 20
DA77803	*S.* Typhimurium	This study	Evolution of DA77267 in MH at generation 20
DA77804	*S.* Typhimurium	This study	Evolution of DA77269 in MH at generation 20
DA77805	*S.* Typhimurium	This study	Evolution of DA77271 in MH at generation 20
DA77806	*S.* Typhimurium	This study	Evolution of DA77273 in MH at generation 20
DA77807	*S.* Typhimurium	This study	Evolution of DA77276 in MH at generation 20
DA77808	*S.* Typhimurium	This study	Evolution of DA77277 in MH at generation 20
DA77809	*S.* Typhimurium	This study	Evolution of DA77282 in MH at generation 20
DA77810	*S.* Typhimurium	This study	Evolution of DA77283 in MH at generation 20
DA77811	*S.* Typhimurium	This study	Evolution of DA77285 in MH at generation 20
DA77843	*E. coli*	This study	Evolution of DA76837 in MH at generation 20
DA77844	*E. coli*	This study	Evolution of DA76838 in MH at generation 20
DA77845	*E. coli*	This study	Evolution of DA76839 in MH at generation 20
DA77846	*E. coli*	This study	Evolution of DA76840 in MH at generation 20
DA77847	*E. coli*	This study	Evolution of DA76841 in MH at generation 20
DA77848	*E. coli*	This study	Evolution of DA76842 in MH at generation 20
DA77849	*E. coli*	This study	Evolution of DA76843 in MH at generation 20
DA77850	*E. coli*	This study	Evolution of DA76844 in MH at generation 20
DA77851	*K. pneumoniae*	This study	Evolution of DA76845 in MH at generation 20
DA77852	*K. pneumoniae*	This study	Evolution of DA76846 in MH at generation 20
DA77853	*K. pneumoniae*	This study	Evolution of DA76847 in MH at generation 20
DA77854	*K. pneumoniae*	This study	Evolution of DA76848 in MH at generation 20
DA77855	*S.* Typhimurium	This study	Evolution of DA76849 in MH at generation 20
DA77856	*S.* Typhimurium	This study	Evolution of DA76850 in MH at generation 20
DA77857	*S.* Typhimurium	This study	Evolution of DA76851 in MH at generation 20
DA77858	*S.* Typhimurium	This study	Evolution of DA76852 in MH at generation 20
DA77859	*E. coli*	This study	Evolution of DA77138 in MH at generation 50
DA77860	*E. coli*	This study	Evolution of DA77140 in MH at generation 50
DA77861	*E. coli*	This study	Evolution of DA77142 in MH at generation 50
DA77862	*E. coli*	This study	Evolution of DA77145 in MH at generation 50
DA77863	*E. coli*	This study	Evolution of DA77146 in MH at generation 50
DA77864	*E. coli*	This study	Evolution of DA77148 in MH at generation 50
DA77865	*E. coli*	This study	Evolution of DA77150 in MH at generation 50
DA77866	*E. coli*	This study	Evolution of DA77151 in MH at generation 50
DA77867	*E. coli*	This study	Evolution of DA77155 in MH at generation 50
DA77868	*E. coli*	This study	Evolution of DA77158 in MH at generation 50
DA77869	*E. coli*	This study	Evolution of DA77160 in MH at generation 50
DA77870	*E. coli*	This study	Evolution of DA77161 in MH at generation 50
DA77871	*E. coli*	This study	Evolution of DA77162 in MH at generation 50
DA77872	*E. coli*	This study	Evolution of DA77165 in MH at generation 50
DA77873	*E. coli*	This study	Evolution of DA77166 in MH at generation 50
DA77874	*E. coli*	This study	Evolution of DA77169 in MH at generation 50

DA77875	*E. coli*	This study	Evolution of DA77172 in MH at generation 50
DA77876	*E. coli*	This study	Evolution of DA77173 in MH at generation 50
DA77877	*E. coli*	This study	Evolution of DA77175 in MH at generation 50
DA77878	*E. coli*	This study	Evolution of DA77176 in MH at generation 50
DA77879	*E. coli*	This study	Evolution of DA77178 in MH at generation 50
DA77880	*E. coli*	This study	Evolution of DA77180 in MH at generation 50
DA77881	*E. coli*	This study	Evolution of DA77182 in MH at generation 50
DA77882	*E. coli*	This study	Evolution of DA77184 in MH at generation 50
DA77883	*K. pneumoniae*	This study	Evolution of DA77238 in MH at generation 50
DA77884	*K. pneumoniae*	This study	Evolution of DA77239 in MH at generation 50
DA77885	*K. pneumoniae*	This study	Evolution of DA77241 in MH at generation 50
DA77886	*K. pneumoniae*	This study	Evolution of DA77244 in MH at generation 50
DA77887	*K. pneumoniae*	This study	Evolution of DA77245 in MH at generation 50
DA77888	*K. pneumoniae*	This study	Evolution of DA77248 in MH at generation 50
DA77889	*K. pneumoniae*	This study	Evolution of DA77249 in MH at generation 50
DA77890	*K. pneumoniae*	This study	Evolution of DA77250 in MH at generation 50
DA77891	*K. pneumoniae*	This study	Evolution of DA77253 in MH at generation 50
DA77892	*K. pneumoniae*	This study	Evolution of DA77255 in MH at generation 50
DA77893	*K. pneumoniae*	This study	Evolution of DA77257 in MH at generation 50
DA77894	*K. pneumoniae*	This study	Evolution of DA77260 in MH at generation 50
DA77895	*S.* Typhimurium	This study	Evolution of DA77262 in MH at generation 50
DA77896	*S.* Typhimurium	This study	Evolution of DA77263 in MH at generation 50
DA77897	*S.* Typhimurium	This study	Evolution of DA77265 in MH at generation 50
DA77898	*S.* Typhimurium	This study	Evolution of DA77267 in MH at generation 50
DA77899	*S.* Typhimurium	This study	Evolution of DA77269 in MH at generation 50
DA77900	*S.* Typhimurium	This study	Evolution of DA77271 in MH at generation 50
DA77901	*S.* Typhimurium	This study	Evolution of DA77273 in MH at generation 50
DA77902	*S.* Typhimurium	This study	Evolution of DA77276 in MH at generation 50
DA77903	*S.* Typhimurium	This study	Evolution of DA77277 in MH at generation 50
DA77904	*S.* Typhimurium	This study	Evolution of DA77282 in MH at generation 50
DA77905	*S.* Typhimurium	This study	Evolution of DA77283 in MH at generation 50
DA77906	*S.* Typhimurium	This study	Evolution of DA77284 in MH at generation 50
DA77915	*E. coli*	This study	Evolution of DA76837 in MH at generation 50
DA77916	*E. coli*	This study	Evolution of DA76838 in MH at generation 50
DA77917	*E. coli*	This study	Evolution of DA76839 in MH at generation 50
DA77918	*E. coli*	This study	Evolution of DA76840 in MH at generation 50
DA77919	*E. coli*	This study	Evolution of DA76841 in MH at generation 50
DA77920	*E. coli*	This study	Evolution of DA76842 in MH at generation 50
DA77921	*E. coli*	This study	Evolution of DA76843 in MH at generation 50
DA77922	*E. coli*	This study	Evolution of DA76844 in MH at generation 50
DA77923	*K. pneumoniae*	This study	Evolution of DA76845 in MH at generation 50
DA77924	*K. pneumoniae*	This study	Evolution of DA76846 in MH at generation 50
DA77925	*K. pneumoniae*	This study	Evolution of DA76847 in MH at generation 50
DA77926	*K. pneumoniae*	This study	Evolution of DA76848 in MH at generation 50
DA77927	*S.* Typhimurium	This study	Evolution of DA76849 in MH at generation 50
DA77928	*S.* Typhimurium	This study	Evolution of DA76850 in MH at generation 50
DA77929	*S.* Typhimurium	This study	Evolution of DA76851 in MH at generation 50
DA77930	*S.* Typhimurium	This study	Evolution of DA76852 in MH at generation 50
DA77945	*E. coli*	This study	Evolution of DA77138 in MH at generation 100
DA77946	*E. coli*	This study	Evolution of DA77140 in MH at generation 100
DA77947	*E. coli*	This study	Evolution of DA77141 in MH at generation 100
DA77948	*E. coli*	This study	Evolution of DA77145 in MH at generation 100
DA77949	*E. coli*	This study	Evolution of DA77146 in MH at generation 100
DA77950	*E. coli*	This study	Evolution of DA77148 in MH at generation 100
DA77951	*E. coli*	This study	Evolution of DA77150 in MH at generation 100
DA77952	*E. coli*	This study	Evolution of DA77151 in MH at generation 100
DA77953	*E. coli*	This study	Evolution of DA77155 in MH at generation 100
DA77954	*E. coli*	This study	Evolution of DA77158 in MH at generation 100
DA77955	*E. coli*	This study	Evolution of DA77160 in MH at generation 100
DA77956	*E. coli*	This study	Evolution of DA77161 in MH at generation 100
DA77957	*E. coli*	This study	Evolution of DA77162 in MH at generation 100
DA77958	*E. coli*	This study	Evolution of DA77165 in MH at generation 100
DA77959	*E. coli*	This study	Evolution of DA77166 in MH at generation 100
DA77960	*E. coli*	This study	Evolution of DA77169 in MH at generation 100
DA77961	*E. coli*	This study	Evolution of DA77172 in MH at generation 100
DA77962	*E. coli*	This study	Evolution of DA77173 in MH at generation 100
DA77963	*E. coli*	This study	Evolution of DA77175 in MH at generation 100
DA77964	*E. coli*	This study	Evolution of DA77176 in MH at generation 100

DA77965	*E. coli*	This study	Evolution of DA77178 in MH at generation 100
DA77966	*E. coli*	This study	Evolution of DA77180 in MH at generation 100
DA77967	*E. coli*	This study	Evolution of DA77182 in MH at generation 100
DA77968	*E. coli*	This study	Evolution of DA77184 in MH at generation 100
DA77969	*K. pneumoniae*	This study	Evolution of DA77238 in MH at generation 100
DA77970	*K. pneumoniae*	This study	Evolution of DA77239 in MH at generation 100
DA77971	*K. pneumoniae*	This study	Evolution of DA77241 in MH at generation 100
DA77972	*K. pneumoniae*	This study	Evolution of DA77244 in MH at generation 100
DA77973	*K. pneumoniae*	This study	Evolution of DA77245 in MH at generation 100
DA77974	*K. pneumoniae*	This study	Evolution of DA77248 in MH at generation 100
DA77975	*K. pneumoniae*	This study	Evolution of DA77249 in MH at generation 100
DA77976	*K. pneumoniae*	This study	Evolution of DA77250 in MH at generation 100
DA77977	*K. pneumoniae*	This study	Evolution of DA77253 in MH at generation 100
DA77978	*K. pneumoniae*	This study	Evolution of DA77255 in MH at generation 100
DA77979	*K. pneumoniae*	This study	Evolution of DA77257 in MH at generation 100
DA77980	*K. pneumoniae*	This study	Evolution of DA77260 in MH at generation 100
DA77981	*S.* Typhimurium	This study	Evolution of DA77262 in MH at generation 100
DA77982	*S.* Typhimurium	This study	Evolution of DA77263 in MH at generation 100
DA77983	*S.* Typhimurium	This study	Evolution of DA77265 in MH at generation 100
DA77984	*S.* Typhimurium	This study	Evolution of DA77267 in MH at generation 100
DA77985	*S.* Typhimurium	This study	Evolution of DA77269 in MH at generation 100
DA77986	*S.* Typhimurium	This study	Evolution of DA77271 in MH at generation 100
DA77987	*S.* Typhimurium	This study	Evolution of DA77273 in MH at generation 100
DA77988	*S.* Typhimurium	This study	Evolution of DA77276 in MH at generation 100
DA77989	*S.* Typhimurium	This study	Evolution of DA77277 in MH at generation 100
DA77990	*S.* Typhimurium	This study	Evolution of DA77282 in MH at generation 100
DA77991	*S.* Typhimurium	This study	Evolution of DA77283 in MH at generation 100
DA77992	*S.* Typhimurium	This study	Evolution of DA77284 in MH at generation 100
DA78002	*E. coli*	This study	Evolution of DA76837 in MH at generation 100
DA78003	*E. coli*	This study	Evolution of DA76838 in MH at generation 100
DA78004	*E. coli*	This study	Evolution of DA76839 in MH at generation 100
DA78005	*E. coli*	This study	Evolution of DA76840 in MH at generation 100
DA78006	*E. coli*	This study	Evolution of DA76841 in MH at generation 100
DA78007	*E. coli*	This study	Evolution of DA76842 in MH at generation 100
DA78008	*E. coli*	This study	Evolution of DA76843 in MH at generation 100
DA78009	*E. coli*	This study	Evolution of DA76844 in MH at generation 100
DA78010	*K. pneumoniae*	This study	Evolution of DA76845 in MH at generation 100
DA78011	*K. pneumoniae*	This study	Evolution of DA76846 in MH at generation 100
DA78012	*K. pneumoniae*	This study	Evolution of DA76847 in MH at generation 100
DA78013	*K. pneumoniae*	This study	Evolution of DA76848 in MH at generation 100
DA78014	*S.* Typhimurium	This study	Evolution of DA76849 in MH at generation 100
DA78015	*S.* Typhimurium	This study	Evolution of DA76850 in MH at generation 100
DA78016	*S.* Typhimurium	This study	Evolution of DA76851 in MH at generation 100
DA78017	*S.* Typhimurium	This study	Evolution of DA76852 in MH at generation 100
DA78018	*E. coli*	This study	Evolution of DA77138 in MH at generation 150
DA78019	*E. coli*	This study	Evolution of DA77140 in MH at generation 150
DA78020	*E. coli*	This study	Evolution of DA77142 in MH at generation 150
DA78021	*E. coli*	This study	Evolution of DA77145 in MH at generation 150
DA78022	*E. coli*	This study	Evolution of DA77146 in MH at generation 150
DA78023	*E. coli*	This study	Evolution of DA77148 in MH at generation 150
DA78024	*E. coli*	This study	Evolution of DA77150 in MH at generation 150
DA78025	*E. coli*	This study	Evolution of DA77151 in MH at generation 150
DA78026	*E. coli*	This study	Evolution of DA77155 in MH at generation 150
DA78027	*E. coli*	This study	Evolution of DA77158 in MH at generation 150
DA78028	*E. coli*	This study	Evolution of DA77160 in MH at generation 150
DA78029	*E. coli*	This study	Evolution of DA77161 in MH at generation 150
DA78030	*E. coli*	This study	Evolution of DA77162 in MH at generation 150
DA78031	*E. coli*	This study	Evolution of DA77165 in MH at generation 150
DA78032	*E. coli*	This study	Evolution of DA77166 in MH at generation 150
DA78033	*E. coli*	This study	Evolution of DA77169 in MH at generation 150
DA78034	*E. coli*	This study	Evolution of DA77172 in MH at generation 150
DA78035	*E. coli*	This study	Evolution of DA77173 in MH at generation 150
DA78036	*E. coli*	This study	Evolution of DA77175 in MH at generation 150
DA78037	*E. coli*	This study	Evolution of DA77176 in MH at generation 150
DA78038	*E. coli*	This study	Evolution of DA77178 in MH at generation 150
DA78039	*E. coli*	This study	Evolution of DA77180 in MH at generation 150
DA78040	*E. coli*	This study	Evolution of DA77182 in MH at generation 150
DA78041	*E. coli*	This study	Evolution of DA77184 in MH at generation 150

DA78042	*K. pneumoniae*	This study	Evolution of DA77238 in MH at generation 150
DA78043	*K. pneumoniae*	This study	Evolution of DA77239 in MH at generation 150
DA78044	*K. pneumoniae*	This study	Evolution of DA77241 in MH at generation 150
DA78045	*K. pneumoniae*	This study	Evolution of DA77244 in MH at generation 150
DA78046	*K. pneumoniae*	This study	Evolution of DA77245 in MH at generation 150
DA78047	*K. pneumoniae*	This study	Evolution of DA77248 in MH at generation 150
DA78048	*K. pneumoniae*	This study	Evolution of DA77249 in MH at generation 150
DA78049	*K. pneumoniae*	This study	Evolution of DA77250 in MH at generation 150
DA78050	*K. pneumoniae*	This study	Evolution of DA77253 in MH at generation 150
DA78051	*K. pneumoniae*	This study	Evolution of DA77255 in MH at generation 150
DA78052	*K. pneumoniae*	This study	Evolution of DA77257 in MH at generation 150
DA78053	*K. pneumoniae*	This study	Evolution of DA77260 in MH at generation 150
DA78054	*S.* Typhimurium	This study	Evolution of DA77262 in MH at generation 150
DA78055	*S.* Typhimurium	This study	Evolution of DA77263 in MH at generation 150
DA78056	*S.* Typhimurium	This study	Evolution of DA77265 in MH at generation 150
DA78057	*S.* Typhimurium	This study	Evolution of DA77267 in MH at generation 150
DA78058	*S.* Typhimurium	This study	Evolution of DA77269 in MH at generation 150
DA78059	*S.* Typhimurium	This study	Evolution of DA77271 in MH at generation 150
DA78060	*S.* Typhimurium	This study	Evolution of DA77273 in MH at generation 150
DA78061	*S.* Typhimurium	This study	Evolution of DA77276 in MH at generation 150
DA78062	*S.* Typhimurium	This study	Evolution of DA77277 in MH at generation 150
DA78063	*S.* Typhimurium	This study	Evolution of DA77282 in MH at generation 150
DA78064	*S.* Typhimurium	This study	Evolution of DA77283 in MH at generation 150
DA78065	*S.* Typhimurium	This study	Evolution of DA77284 in MH at generation 150
DA78099	*E. coli*	This study	Evolution of DA76837 in MH at generation 150
DA78100	*E. coli*	This study	Evolution of DA76838 in MH at generation 150
DA78101	*E. coli*	This study	Evolution of DA76839 in MH at generation 150
DA78102	*E. coli*	This study	Evolution of DA76840 in MH at generation 150
DA78103	*E. coli*	This study	Evolution of DA76841 in MH at generation 150
DA78104	*E. coli*	This study	Evolution of DA76842 in MH at generation 150
DA78105	*E. coli*	This study	Evolution of DA76843 in MH at generation 150
DA78106	*E. coli*	This study	Evolution of DA76844 in MH at generation 150
DA78107	*K. pneumoniae*	This study	Evolution of DA76845 in MH at generation 150
DA78108	*K. pneumoniae*	This study	Evolution of DA76846 in MH at generation 150
DA78109	*K. pneumoniae*	This study	Evolution of DA76847 in MH at generation 150
DA78110	*K. pneumoniae*	This study	Evolution of DA76848 in MH at generation 150
DA78111	*S.* Typhimurium	This study	Evolution of DA76849 in MH at generation 150
DA78112	*S.* Typhimurium	This study	Evolution of DA76850 in MH at generation 150
DA78113	*S.* Typhimurium	This study	Evolution of DA76851 in MH at generation 150
DA78114	*S.* Typhimurium	This study	Evolution of DA76852 in MH at generation 150

Supplementary Table 7. List of primers used in this study

ddPCR primers

Primer	Sequence	Description
aac(3)-F	AATCCGATGCCGTTTTCCAG	Oligo binding to the putative resistance gene *aac(3)-IId* in the plasmid pDA33135-139
aac(3)-R	AAACTCCGTTACCGCATTGC	
plasmid6-F	CTCCCTGAGAAGAATGGCCA	Oligo binding to the plasmid pDA33135-139 outside amplification region
plasmid6-R	GCATATGGTGACGCTGATCC	
fumD-F	AAATGTGCAGAGTTGTCGGT	Oligo binding to the chromosome DA33135
fumC-R	CTGTTTCCATCGCCTGCTTT	
aac(3)-IId-F	AAACTCCGTTACCGCATTGC	Oligo binding to the putative resistance gene *aac(3)-IId* in the plasmid pDA33137-178
aac(3)-IId-R	TCACCGTCTCTTCCAAGCAT	
ssb-F	CTTACGTCCACGGCCTTTC	Oligo binding to the plasmid pDA33137-178 outside amplification region
ssb-R	GCAACAGTTCAGCGGTCAG	
cysG-F	TTGTCGGCGGTGGTGATGTC	Oligo binding to the chromosome DA33137
cysG-R	ATGCGGTGAACTGTGGAATAAACG	
aac(3)-IIa-F	GCATGCCTCACTTAAAGCGA	Oligo binding to the putative resistance gene *aac(3)-IIa* in the plasmid pDA33140-96
aac(3)-IIa-R	TATCCCATCACAGTGCCAGT	
repB-F	GCGCGAACGATAGTGGTATT	Oligo binding to the plasmid pDA33140-96 outside amplification region
repB-R	ACTTGCGAACAAAGGGACAG	
dnaK-F	CCAGCTCTTCGAACTTACGG	Oligo binding to the chromosome DA33140
dnaK-R	ATCAAAGCGTCTTCCGGTCT	
tetA-F	GCGGTCGGTATTGTCTTCAC	Oligo binding to the putative resistance gene *tet(A)* in the chromosome DA34827
tetA-R	GGATGCAGAAGTAGAACGCG	
hcaT-F	ACGATTACCAGGCGATTCTG	Oligo binding to the chromosome DA34827
hcaT-R	CGCTGGGACGTAACAACATAC	

Strain reconstruction primers

Primer	Sequence	Description
cyoB-R71G-P1	GCCTCGGTATCATGTATATCATCGTGGCGATTGTGATGTTGCTGgtgtaggctggagctgcttc	Primers for amplification of the *cat-sacB*-YFP cassette with flanking homologies to the target region
cyoB-R71G-P2	CAAGAGCCTGCTGGCTACGCATCATAATGGCGTCAGCAAAACCcatatgaatatcctccttagttcc	
cyoB-R71G-ss	GCCTCGGTATCATGTATATCATCGTGGCGATTGTGATGTTGCTGGGTGGTTTTGCTGACGCCATTATGATGCGTAGCCAGCAGGCTCTTG	Oligos for replacing the *cat-sacB*-YFP cassette and introducing the point mutation
cyoB-Fwd	CCAACGTGAAACCAGACTTGT	Primers for PCR and sequencing of the gene locus with mutation
cyoB-Rev	CGTCAGAATTGGGAAGGAAGC	
nuoG-F33V-P1	GCTGGAAGCTTGTCTGTCTCTGGGCCTTGATATTCCTTACgtgtaggctggagctgcttc	Primers for amplification of the *cat-sacB*-YFP cassette with flanking homologies to the target region
nuoG-F33V-P2	CTGGCGGCAAGCACCGACACTTCCCAGCGCCGGATGCCAGCAcatatgaatatcctccttagttcc	
nuoG-F33V-ss	CTGGCGGCAAGCACCGACACTTCCCAGCGCCGGATGCCAGCAAACGTAAGGAATATCAAGGCCCAGAGACAGACAAGCTTCCAGC	Oligos for replacing the *cat-sacB*-YFP cassette and introducing the point mutation
nuoG-Fwd	CGTGAAAATTCTGCGTGCG	Primers for PCR and sequencing of the gene locus with mutation
nuoG-Rev	CGACCGAAGTAGACGTTGTC	
tetR/acrR-L179P-P1	GCCGCGCGCGATCCCGCTCGCGCTGGCGAATATATTGCGgtgtaggctggagctgcttc	Primers for amplification of the *cat-sacB*-YFP cassette with flanking homologies to the target region
tetR/acrR-L179P-P2	GCTCCTCGCGAGTCAGCGCGCGCCACATGGCTTCGAATCCcatatgaatatcctccttagttcc	
tetR/acrR-L179P-ss	GCCGCGCGCGATCCCGCTCGCGCTGGCGAATATATTGCGCCGGGATTCGAAGCCATGTGGCGCGCGCTGACTCGCGAGGAGC	Oligos for replacing the *cat-sacB*-YFP cassette and introducing the point mutation
tetR/acrR-Fwd	TTTCGCGACCAAAGATGAGC	Primers for PCR and sequencing of the gene locus with mutation
tetR/acrR-Rev	AGGGAGGAAAATGCGGAAAC	
rcsB-P60L-P1	CCGAAATTAGATGCGCATGTGTTGATCACTGACCTCTCCATGgtgtaggctggagctgcttc	Primers for amplification of the *cat-sacB*-YFP cassette with flanking homologies to the target region
rcsB-P60L-P2	GCTTGATGTACTTGATCAAGGTGATCCCATCGCCGTATTTATCTCCcatatgaatatcctccttagttcc	
rcsB-P60L-ss	CCGAAATTAGATGCGCATGTGTTGATCACTGACCTCTCCATGCTGGGAGATAAATACGGCGATGGGATCACCTTGATCAAGTACATCAAGC	Oligos for replacing the *cat-sacB*-YFP cassette and introducing the point mutation
rcsB-Fwd	ATTATTGCCGATGACCACCC	Primers for PCR and sequencing of the gene locus with mutation
rcsB-Rev	GCGATTTATTCTTTGTCTGTCGG	
